# Genome-wide association tests by two-stage approaches with unified analysis of families and unrelated individuals

**DOI:** 10.1186/1753-6561-1-s1-s140

**Published:** 2007-12-18

**Authors:** Xuexia Wang, Zhaogong Zhang, Shuanglin Zhang, Qiuying Sha

**Affiliations:** 1 Department of Mathematical Sciences, Michigan Technological University, 1400 Townsend Drive, Houghton, Michigan 49931, USA; 2 Department of Mathematics, Heilongjiang University, 74 Xuefu Road, Harbin 150080, China

## Abstract

Multiple testing is a problem in genome-wide or region-wide association studies. In this report, we consider a study design given by the Genetic Analysis Workshop 15 (GAW15) Problem 3 – nuclear families (parents with their affected children) and unrelated controls. Based on this design, we propose three two-stage approaches to deal with the problem of multiple testing. The tests in the first stage, statistically independent of the association test used in the second stage, are used to screen or select single-nucleotide polymorphisms (SNPs). Then, in the second stage, a family-based association test is performed on a much smaller set of selected SNPs. Thus, the problem of multiple testing is much less severe. Our simulation studies and application to the dense SNP data of chromosome 6 in the GAW15 Problem 3 show that the two-stage methods are more powerful than the one-stage method (using the family-based association test only).

## Background

Genome-wide or region-wide association is a promising approach to mapping complex disease genes [1,2]. However, the success of genome-wide or region-wide association studies will depend on whether the information gain of increased number of single-nucleotide polymorphisms (SNPs) will be diluted by the multiple-comparison problem [3]. When tens or hundreds of thousands of SNPs are tested for association, the *p*-values need to be adjusted for controlling type I error rates. Most multiple-testing adjustment approaches, including Bonferroni correction for controlling the family-wise error rate and the method proposed by Benjamini and Hochberg [4] for controlling the false discovery rate (FDR), become more conservative as more tests are done.

In case-control studies, several authors have proposed a two-stage design that utilizes two independent samples [5,6]. The first sample is used to screen and select SNPs for association tests. The association tests are conducted on the selected SNPs by using the second sample, so that the number of association tests is diminished and the correction for multiple testing is less severe. Recently, in mapping quantitative trait loci using family data, Van Steen et al. [3] proposed an interesting approach that performs the SNP screening and association test using the same sample. The basic idea of Van Steen et al.'s method is that the screening test based on the traits and between-family genotype scores is statistically independent of the association test that depends on trait values and within-family genotype scores. The screening test is used first to select SNPs, and the association test is performed on a much smaller set of selected SNPs. Unfortunately, the same idea cannot be applied to family-based analyses for qualitative traits.

In this article, we propose several two-stage methods to test association for qualitative traits by using nuclear families (including parental phenotypes) or nuclear families and unrelated controls. To analyze the data set of nuclear families, we compare the allele frequency in affected parents with that of unaffected parents (test I) to screen and select SNPs. Then the pedigree disequilibrium test (PDT) [7] is used to perform the association test on the selected SNPs by comparing the alleles that are transmitted to the children with those that are not transmitted. To analyze the data set that contains nuclear families and unrelated controls, as the data set in the GAW15 Problem 3, we propose two methods to screen SNPs. One is comparing the allele frequency in parents with that in unrelated controls (test II). The other is a combination of test I and II. All the proposed screening tests are independent of the association test, that is, the PDT. Furthermore, because a significant association only depends on the results of the PDT, the proposed two-stage approaches are robust to population admixture. We compare the performance of the proposed methods by using PDT alone through simulation studies and analysis of the data set of the GAW15 Problem 3. Our simulation and the GAW15 data analysis results show that the three proposed two-stage methods have correct type I error rates and, in most cases, are more powerful than the PDT.

## Methods

Consider a sample of *n *nuclear families and *N *unrelated controls. Suppose that we have genotyped *M *markers across the genome or in a candidate region for each sampled individual. Also, all children in the nuclear families are affected and the disease status of the parents is available. The reason for considering this kind of sample is the design of the GAW15 Problem 3 data set. To detect disease susceptibility loci, based on the sample structure, we proposed three methods. All three methods are two-stage approaches – the methods in the first stage are used to screen and select SNPs and those in the second stage are used to test the association on the selected SNPs.

The three approaches we propose for the first stage are based on a test statistic for a case-control study. Consider a case-control study with *N*_1_ cases and *N*_2_ controls, and each sampled individual has a genotype at a bi-allelic marker with two alleles A and a. To test the association between the marker and the disease, one can use the statistic:

(1) $$T=\frac{\hat{p}-\hat{q}}{\sigma },$$

where $\hat{p}$ and $\hat{q}$ are the sample frequencies of allele A in cases and controls, respectively; $\sigma^{2}=(\frac{1}{2N_{1}}+\frac{1}{2N_{2}})p_{0}(1-p_{0})$ is the estimate of the variance of $\hat{p}$ - $\hat{q}$; *p*_0 _is the sample allele frequency of allele A in the whole sample. Under the null hypothesis of no association, this test statistic asymptotically follows a standard normal distribution. When the absolute value of *T *is large, we reject the null hypothesis of no association. Based on the test statistic *T*, we propose the following three tests that can be used in the first stage to screen SNPs:

1. Consider affected parents of the sampled nuclear families as cases and unaffected parents of the sampled nuclear families as controls. The test statistic *T *based on this sample is denoted by *T*_*cc*_. The *T*_*cc *_only uses the nuclear families (does not need the unrelated controls).

2. Consider all the parents of the *n *sampled nuclear families as cases and the *N *sampled unrelated controls as controls. The test statistic *T *based on this sample is denoted by *T*_*pc*_. If A is a high risk allele, the frequency of A among the parents should be higher than that in the controls, because each pair of parents has at least one affected child.

3. The third approach is a combination of the *T*_*pc *_and *T*_*cc*_. The test statistic of this approach is Fisher's combination of the *p*-values of the two tests and is given by *T*_*cb *_= -2(log *P*_1 _+ log *P*_2_), where *P*_1 _and *P*_2 _are the *p*-values of the tests *T*_*pc *_and *T*_*cc*_, respectively. Under the null hypothesis of no association, *T*_*cb *_will follow a *χ*^2 ^distribution with 4 degrees of freedom [8].

We use the PDT [7] to test association in the second stage. Suppose there are *n*_*i *_affected children in the *i*^th ^family.For a biallelic marker with two alleles A and a, we code the three genotypes aa, Aa, and AA as 0, 1, and 2, respectively. Let *X*_*ij*_, *X*_*iF*_, and *X*_*iM *_denote the codes of the genotypes of the *j*^th ^child, father, and mother in the *i*^th ^family. Let $U_{i}=\frac{1}{n_{i}}\sum_{j=1}^{n_{i}}\left(X_{ij}-\frac{X_{iF}+X_{iM}}{2}\right)$, $U=\sum_{i=1}^{n}U_{i}$, and ${\hat{\sigma }}^{2}=\sum_{i=1}^{n}U_{i}^{2}$.

Then the test statistic of the PDT is given by $PDT=U/\hat{\sigma }$. Under null hypothesis of no association, the PDT follows the standard normal distribution.

When we apply the two-stage approaches, we first apply one of *T*_*pc*_, *T*_*cc*_, or *T*_*cb *_to each of the *M *markers and get *M p*-values. Select *L *markers with the smallest *p*-values (we will discuss later how to choose *L*). Then, we apply the PDT to the *L *selected SNPs, and declare a SNP as significant if the *p*-value of the PDT at this marker is less than a threshold *δ*_*Lα *_. The threshold *δ*_*Lα *_is determined by controlling the FDR, the ratio of the number of falsely rejected null hypotheses to the total number of rejected null hypotheses, at level *α*. To control the FDR we can choose the cut-off *δ*_*Lα *_as follows [4]: let *p*_(1)_,...,*p*_(*L*) _be the ordered *p*-values when we apply the PDT to the *L *selected markers, then $\delta_{L\alpha }=\max\{p_{\left(i\right)}:p_{\left(i\right)}\leq \frac{i\alpha }{L}\}$.

In our simulation studies and application to analyze the GAW15 simulated data, we use the following method to calculate the power of the two-stage test to detect one disease locus, say locus D. Suppose that there are *K *replicated samples. Let *k *denote the number of samples in which locus D is selected in the first stage and the *p*-value of locus D in the second stage is less than *δ*_*Lα *_. Then, the power to detect Locus D is *k/K*.

## Results

### Simulated data

We first evaluate the FDR under null the hypothesis of no marker associated with disease. Under the null hypothesis, the FDR and the family-wise type I error rate will be the same. We also evaluate the statement of the independence between the tests in the first stage and the PDT in the second stage. For these purposes, we generate genotypes for each individual at 100 SNPs. For a given rare allele frequency, we generate the genotypes of the parents of the nuclear families and unrelated controls by assuming Hardy-Weinberg equilibrium and independence between markers. Each of the parents randomly transmits one of the two alleles to a child to form the child's genotype. We consider two different sample sizes, two different rare allele frequencies, and two different numbers of children in each family. For each simulation scenario, we generate 1000 samples to estimate the FDR at nominal level 0.05. For each of the two-stage approaches, we use a threshold *α *to select SNPs in the first stage, that is, we select all SNPs whose *p*-values are less than *α*. We choose several values for the threshold. If a two-stage approach has a correct FDR, we know that the tests used in the first stage and in the second stage should be independent. The results are summarized in Table 1. Table 1 shows that the FDRs of the three two-stage approaches are very consistent with the nominal level, especially when the sample size is large or the minor allele frequency is large (>0.1). The FDR of the one-stage test PDT is slightly conservative for a small sample size. When the sample size is large (>100 families and 120 controls), the FDRs of the PDT are also very consistent with the nominal level.

**Table 1:** Estimated type I error rates at nominal level 0.05, 1000 replicate simulation data sets

			Two children in each family^b^				One child in each family^b^			
				
Sample size	Minor allele frequency	*α*^a^	PDT	*T*_*pc*_	*T*_*cc*_	*T*_*cb*_	PDT	*T*_*pc*_	*T*_*cc*_	*T*_*cb*_
100 families, 120 controls	0.1	0.05	0.014	0.032	0.043	0.042	0.031	0.033	0.044	0.043
		0.1		0.023	0.028	0.032		0.029	0.041	0.029
		0.2		0.025	0.027	0.027		0.031	0.031	0.026
										
	0.3	0.05	0.024	0.042	0.049	0.047	0.036	0.05	0.045	0.053
		0.1		0.04	0.043	0.035		0.051	0.047	0.046
		0.2		0.046	0.038	0.037		0.054	0.047	0.053
										
500 families, 600 controls	0.1	0.05	0.048	0.051	0.051	0.059	0.053	0.052	0.054	0.051
		0.1		0.04	0.052	0.042		0.052	0.041	0.045
		0.2		0.042	0.046	0.044		0.04	0.047	0.05
										
	0.3	0.05	0.059	0.053	0.047	0.045	0.042	0.046	0.04	0.042
		0.1		0.054	0.042	0.046		0.048	0.034	0.044
		0.2		0.053	0.034	0.048		0.047	0.043	0.041

^a^*α*, the cut-off *p*-value of the test in the first stage

^b^PDT, pedigree disequilibrium test; *T*_*pc*_, test comparing all parents to controls; *T*_*cc*_, test comparing affected and unaffected parents; *T*_*cb*_, combined *T*_*pc *_and *T*_*cc*_

### GAW15 data analysis

We applied three two-stage approaches and the PDT to analyze the dense SNP data of chromosome 6 in the GAW15 Problem 3 (simulated rheumatoid arthritis data). The data contain 100 replicate data sets. In each replicate data set, there are 1500 nuclear families, 2000 unrelated controls and two affected children in each family. Each individual has genotypes at 17,820 SNPs on chromosome 6. From the answer provided with the data set, we know that there are three trait loci: Locus DR, Locus C, and Locus D on chromosome 6. Locus DR affects the risk of rheumatoid arthritis (RA). Locus C increases RA risk only in women. These two loci are in the same position. The typed SNP 3437 on chromosome 6 is in the same position as Locus DR and Locus C, that is, the recombination rates between SNP 3437 and Locus DR and between SNP 3437 and Locus C are both zero. The rare allele of Locus D increases RA risk five-fold. In the dense SNP panel of chromosome 6, the SNP that is nearest to Locus D is SNP 3917. The genetic distance between locus D and SNP 3917 is 0.00171 cM, and the physical distance is 1565 bp.

We first compare the power of the four methods to detect SNP 3437. The association between SNP 3437 and RA turns out to be very strong, such that the power of the four methods is all 100%. In order to do the power comparison, we reduce the sample size and, instead of detecting SNP 3437, we detect SNP 3455. SNP 3437 and SNP 3455 are in linkage disequilibrium with *r *= 0.36 and *D*' = 0.45. The results of the power comparison for detecting SNP 3455 using a smaller sample size are summarized in Table 2. The results show that the two-stage approaches *T*_*pc *_and *T*_*cb*_, which use both family data and unrelated controls, are more powerful than the PDT, especially when *α *is 1%, 5%, or 10%. However, the two-stage approach *T*_*cc *_is not as powerful as the PDT when *α *is less than 0.3. When *α *is between 0.5 and 0.8, the *T*_*cc *_is slightly more powerful than the PDT (results not shown).

**Table 2:** Power for detecting SNP 3455 based on the dense SNP data of chromosome 6 by using 150 families and 200 unrelated controls

	Two children in each family^b^				One child in each family^b^			
		
*α*^a^	PDT	*T*_*pc*_	*T*_*cc*_	*T*_*cb*_	PDT	*T*_*pc*_	*T*_*cc*_	*T*_*cb*_
0.01		0.93	0.15	0.96		0.66	0.13	0.87
0.05		0.95	0.39	0.96		0.65	0.29	0.8
0.1	0.83	0.96	0.54	0.96	0.53	0.64	0.4	0.75
0.3		0.91	0.77	0.91		0.58	0.53	0.61
0.5		0.88	0.81	0.88		0.56	0.54	0.56

^a^*α*, the cut-off *p*-value of the test in the first stage

^b^PDT, pedigree disequilibrium test; *T*_*pc*_, test comparing all parents to controls; *T*_*cc*_, test comparing affected and unaffected parents; *T*_*cb*_, combined *T*_*pc *_and *T*_*cc*_

The power of the four methods to detect SNP 3917 is summarized in Table 3. Because the power of all four methods is not very high, we doubled the sample size by merging two replicate data sets together as one sample. The results in Table 3 show that, in the case of one child in each family, all three two-stage approaches are more powerful than the PDT when *α *> 0.05. When the value of *α *is between 0.05 and 0.1, the *T*_*cb *_is much more powerful than the PDT. In the case of two children in each family, the *T*_*cb *_is also more powerful than the PDT when *α *> 0.01. The other two two-stage approaches, the *T*_*pc *_and *T*_*cc*_, are only slightly more powerful than the PDT when *α *≥ 0.05.

**Table 3:** Power for detecting marker 3917 based on the dense SNP data of chromosome 6 by merging two replicate data sets to increase sample size

	Two children in each family^b^				One child in each family^b^			
		
*α*^a^	PDT	*T*_*pc*_	*T*_*cc*_	*T*_*cb*_	PDT	*T*_*pc*_	*T*_*cc*_	*T*_*cb*_
0.01		0.36	0.22	0.44		0.24	0.18	0.32
0.05		0.56	0.32	0.68		0.4	0.28	0.52
0.1	0.56	0.54	0.42	0.68	0.3	0.42	0.36	0.54
0.3		0.58	0.54	0.64		0.32	0.28	0.32
0.5		0.62	0.56	0.64		0.34	0.28	0.34

^a^*α*, the cut-off *p*-value of the test in the first stage

^b^PDT, pedigree disequilibrium test; *T*_*pc*_, test comparing all parents to controls; *T*_*cc*_, test comparing affected and unaffected parents; *T*_*cb*_, combined *T*_*pc *_and *T*_*cc*_

## Discussion

In this report for genome-wide or region-wide association studies, we proposed three two-stage approaches to analyze family data or data sets that contain family data as well as unrelated controls. Based on our simulation studies and applications of the data sets of the GAW15 Problem 3, we are able to demonstrate that, in the case of one child in each family – the typical data set of the TDT design – all three two-stage approaches are more powerful than the PDT. In almost all the cases we considered, the *T*_*cb *_using family data and unrelated controls is more powerful than the PDT, and in several cases the *T*_*cb *_can double the power of the PDT. How to choose the value of the threshold *α *is a problem. From our simulation studies, one can see that the value of *α *around 0.01 may be a good choice for the *T*_*pc *_and *T*_*cb*_. If only the family data are available, we would use the two-stage approach *T*_*cc*_. In the case of one child, the value around 0.1 may be a good choice for *α*. In the case of two children, the *T*_*cc *_does not benefit much. In general, we need further investigation for choosing the value of *α*.

## Conclusion

Our simulation and the GAW15 data analysis results show that the three proposed two-stage methods have correct type I error rates and, in most cases, are more powerful than the PDT.

## Competing interests

The author(s) declare that they have no competing interests.
